# Cytotoxic, Antimitotic, DNA Binding, Photocatalytic, H_2_O_2_ Sensing, and Antioxidant Properties of Biofabricated Silver Nanoparticles Using Leaf Extract of *Bryophyllum pinnatum* (Lam.) Oken

**DOI:** 10.3389/fmolb.2020.593040

**Published:** 2021-01-28

**Authors:** Sandip Kumar Chandraker, Mishri Lal, Preeti Dhruve, Rana P. Singh, Ravindra Shukla

**Affiliations:** ^1^Laboratory of Bio-Resource Technology, Department of Botany, Indira Gandhi National Tribal University, Amarkantak, India; ^2^Cancer Biology Laboratory, School of Life Sciences, Jawaharlal Nehru University, New Delhi, India

**Keywords:** *Bryophyllum pinnatum*, DNA binding, green synthesis, photocatalyst, cytotoxicity

## Abstract

*Bryophyllum pinnatum* is a perennial herb traditionally used in ethnomedicine. In the present report, silver nanoparticles (AgNPs) were synthesized using *B. pinnatum* leaf extract. BP-AgNPs were confirmed following UV-Vis spectroscopy with SPR peak at 412 nm and further characterized by FTIR, XRD, SEM-EDX, and TEM. Microscopic images confirmed the spherical shape and ~15 nm average size of nanostructures. BP-AgNPs were evaluated for photocatalytic degradation of hazardous dyes (methylene blue and Rhodamine-B) and showed their complete reduction within 100 and 110 min., respectively. BP-AgNPs have emerged as a unique SPR-based novel sensor for the detection of H_2_O_2_, which may deliver exciting prospects in clinical and industrial areas. DPPH and ABTS free radical scavenging activity were studied with respective IC_50_ values of 89 and 259 μg/mL. A strong intercalating interaction of CT-DNA with BP-AgNPs was investigated. Observed chromosomal abnormalities confirm the antimitotic potential of BP-AgNPs in the meristematic root tip. The cytotoxicity of BP-AgNPs against B16F10 (melanoma cell line) and A431 (squamous cell carcinoma cell line), was assessed with respective IC_50_ values of 59.5 and 96.61 μg/ml after 24 h of treatment. The presented green synthetic approach provides a novel and new door for environmental, industrial, and biomedical applications.

## Introduction

The distinctive features of metal nanoparticles (NPs) have made it attractive to various applications such as optical, electronic, magnetic, and antimicrobial for the last few years. (Punjabi et al., 2018; Arun et al., 2019; Guilger-Casagrande and de Lima, 2019; de Oca-Vásquez et al., 2020). NPs are usually synthesized with distinct physical, chemical, and biological methods, but are inconvenienced by their cost, slow process as well as generation of waste causing environmental pollution and biotic harmfulness (Wang et al., 2016; Unni et al., 2017; Gahlawat and Choudhury, 2019; Hamida et al., 2020; Tortella et al., 2020). The physical methods utilize costly equipments and produce unstable larger nanoparticles, while hydrazine and sodium borohydride like hazardous materials are used as reducing agents in the chemical approaches (Mohanta et al., 2017; Chandraker et al., 2019a). Likewise, cautious isolation of bacteria, fungi, and the management of their non-contaminated culture or their byproducts are tedious issues with the microbial synthesis of NPs (Chandraker et al., 2019b).

Green synthesis using plant extracts is a blameless, facile, rapid, reliable, cost-effective, and eco-friendly approach that gives higher stability and better physicochemical characteristics to NPs (Ravi et al., 2013; Kumari et al., 2020). The phytochemicals and secondary metabolites found in plant extracts act as capping agent, and responsible for the reduction of metal ions and formation of NPs. Green synthesized metal/metal oxide NPs like CuO, FeO, and MgO are being utilized enormously in biomedical and clinical applications (Kumari et al., 2018; Sheel et al., 2020; Verma et al., 2020). Instead, AgNPs are preferred over other noble metal NPs like Au, Pt, Pd, Cu, Fe, etc., due to their extensive uses in bioengineering, cosmetics, food packaging, catalysis, electrochemistry, environmental remediation, and pharmaceutical industries as antiseptic agents (Paul et al., 2018; Mohan et al., 2020; Yousaf et al., 2020). Phytochemicals present in the plant extract are capable of capping and reducing Ag^+^ to Ag^0^ (Das et al., 2020). It is, therefore, suitable for large scale fabrication of AgNPs in non-aseptic environments.

The leading mortality rate for cancer is predicted to reach up to 21 million worldwide over the next decade (Siegel et al., 2016). Chemotherapy is a prominent approach of treatment which includes numerous cytotoxic drugs to arrest cancer cells, however, these drugs also harm normal body cells and causes adverse physiological effects (Iqbal et al., 2017). The antagonistic effects can be reduced by using metallic NPs in controlled targeted drug delivery, without affecting the normal cells. The AgNPs are playing an important role in the quality enhancement of anti-cancer drugs with maximum therapeutic effects (Lohcharoenkal et al., 2014; Verma et al., 2019). The cytotoxic agents may interact with the DNA of target cells and disrupt the mitosis and cell cycle. To explore the intracellular action of NPs, studies on their binding with DNA have become a primary interest in recent years (Ribeiro et al., 2018). Apart from some complicated techniques like gel electrophoresis and other electrochemical detection, UV-vis spectroscopy has been established as a convenient, accurate and extremely sensitive mode to illustrate the interaction of NPs with nucleic acids (Komal and Kaushik, 2019).

Removal of water pollutants is a serious concern of researchers worldwide (Yang et al., 2017). Synthetic dyes are one of the chief pollutants as well as hazardous and carcinogenic substances which are processed and released from the paint, fabrics, rubber, leather, paper, cosmetics, and plastic industries. Their catalytic degradation has received widespread attention because of being quick, extremely efficient (as compared to other approaches), and cost-effective approach, which does not trigger secondary contamination.

Hydrogen peroxide (H_2_O_2_) is a strong oxidizer, frequently used in various manufacturing and food processing industries. Despite, its exposure in industrial processes results in various human health risks and environmental issues because of having toxic effects (Tagad et al., 2013). As per US-EPA, it is detrimental to human and aquatic bodies above permissible limit of 30 PPM in potable water (Bhagyaraj and Krupa, 2020). Therefore, a sensitive, accurate and easy detection method for H_2_O_2_ contaminants is highly required.

*Bryophyllum pinnatum* (Lam.) Oken (family: Crassulaceae) is a perennial herb extensively grown in India, China, Tropical African and American countries, and Australia, and used widely in ethnomedicine. The natural succulent leaves of the plant have a range of therapeutic properties, including antibacterial, hypotensive, gastroprotective, immunomodulator, antidiabetic, anti-inflammatory, and anti-leishmania (Chibli et al., 2014). The plant often reported treating insect bites, kidney stones, boils, burns, gastric ulcers, sores, and eye infections, etc. (Fernandes et al., 2019). In this work, we aimed to synthesize AgNPs using leaf extract of *B. pinnatum* and explore their potential for photocatalytic, DNA binding, H_2_O_2_ sensing, free radical scavenging, antimitotic and cytotoxic activities.

## Experimental Section

### Materials

The leaves of *B. pinnatum* were collected during January 2019 near the Kapil Dhara fall of the Amarkantak region under the Anuppur district of Madhya Pradesh, India. Plant species authentication was carried out by the subject experts, and the voucher specimen (DOB/19/BP/044) was deposited in the herbarium of Botany department, IGNTU, Amarkantak, India.

### Chemicals

Silver nitrate (AgNO_3_; AR grade), calf thymus DNA sodium salt (CT-DNA), 2,2-diphenyl-1-picrylhydrazyl (DPPH) and 2,2'-azino-bis(3-ethylbenzothiazoline-6-sulfonic acid) (ABTS) were purchased from Himedia Laboratories Pvt. Ltd., Mumbai, India. Acetocarmine, Hydrogen peroxide (H_2_O_2_), Methylene blue, and Rhodamine-b were obtained from CDH Laboratories Pvt. Ltd. Mumbai.

### Preparation of Leaf Extract

The collected fresh leaves were washed thoroughly twice with double distilled water (DDW) to eliminate dust and natural impurities. The *B. pinnatum* leaf extract (BPLE) was made with 25 g of fresh leaves, which were cut into equal size. The sliced leaves were boiled in 100 mL of DDW for 20 min at 65°C, and after cooling, the leaves extract was filtered twice with whatman No. 1 filter paper and kept at 4°C.

### Phytochemical Analysis

Preliminary phytochemical screening was carried out using the standard protocol followed by Ali et al. (2018) and Chandraker et al. (2020) to identify the phytoconstituents present in BPLE.

### Green Synthesis of BP-AgNPs and Optimization

Respective volumes (0.5, 1.0, 1.5, and 2.0 mL) of BPLE were added individually to 24.5, 24.0, 23.5, and 23 mL of 1 mM AgNO_3_ solutions for a final volume of 25.0 mL in 50 mL Erlenmeyer flasks. Similarly, 23 mL solutions of AgNO_3_ were arranged with four concentrations (0.1, 0.25, 0.5, and 1 mM) and mixed with 2.00 mL BPLE. Further, the effect of pH on the synthesis of BP-AgNPs was analyzed. For this study, 2 mL of BPLE was separately added to 23 mL AgNO_3_ (1 mM) having variable pH viz., 2, 4, 6, and 8. The pH was adjusted using sulfuric acid, and sodium hydroxide solutions drop by drop. To evaluate the effect of temperature on the green synthesis of BP-AgNPs, the reaction mixture was kept at variable temperatures (27, 40, 60, and 80°C) in a hot air oven. The color shift from colorless solution to yellowish/dark brown solution suggesting BP-AgNPs synthesis and confirmed the reduction from Ag^+^ to Ag^0^.

### Characterization of BP-AgNPs

The synthesis of BP-AgNPs were determined by UV-visible spectroscopy (Shimadzu UV-1800). Fourier Transform Infra-Red spectra obtained with FTIR (Bruker, Germany. Model: Vertex 70) using KBr at a resolution of 0.5 cm^−1^ in the diffuse reflectance mode and within a range of 500–4,000 cm^−1^. Studies of X-Ray Diffraction (XRD) were performed using Bruker D8 at 30 kV and 20 mA current with Cu K (I = 1.54 A) to test the crystallinity of BP-AgNPs. Using Scanning electron microscopy (EVO 18; Carl Zeiss, Germany), the surface morphology and elemental composition (Energy Dispersive X-ray Analysis) of BP-AgNPs were determined. Transmission electron microscopy (Technai G20 FEI) operated at 200 kV, and a 104 beam current was used to determine the size of NPs.

### Applications of BP-AgNPs

#### Cytotoxic Activity

Cells were cultured in Dulbecco's Modified Eagle's (DME) medium supplemented with 10% FBS and 1% antibiotic and antimycotic solution at 37°C with 5% CO_2_. For the control group, cells were cultured in medium containing 0.1% DMSO. For the treatment group, cells were incubated with different nanoparticle dispersed concentrations in DMSO (0.1%). The cytotoxicity of BP-AgNPs against A431 (squamous cell carcinoma cell line) and B16F10 (melanoma cell line) was determined using MTT assay (Singh et al., 2013). Both types of cells were seeded at 5,000 cells/well separately in 96-well plates. After 24 h of seeding, cells were incubated with different concentrations of nanoparticles (10, 25, 50, and 100 μg/ml) for 24 and 48 h. On completion of the treatment time, the media was taken out, and the MTT solution was added to each well and incubated for 4 h. Subsequently, 100 μl of DMSO was added to dissolve the formazan crystals formed. Finally, after incubating the plate at 37°C for 10 min, absorbance was recorded on a microplate reader at 570 nm. Using the following formula percent cell viability was calculated:

$$\begin{array}{l} \%\;Cell\;viability\\ =\;\left(\frac{Average\;absorbance\;of\;the\;treated\;sample}{Average\;absorbance\;of\;the\;control\;sample}\right)\times 100 \end{array}$$

#### Impact on Mitosis in the Onion Root Tip

Onion (*Allium cepa*; 2n = 16) bulb was purchased from the Lalpur market in front of IGNTU, Amarkantak. The outer scale of bulbs was cleaned, and the bulbs were placed on 100 mL beaker at room temperature (30 ± 1°C) for root germination (Chandraker et al., 2014). Root tips (2–3 cm) were treated with 20 and 50% of BP-AgNPs suspension for 6 and 12 h, separately. However, DDW was used as a control (Ghosh et al., 2020). After that, roots were submerged in 1 M HCl solution and heated for 4–5 min and transferred to DDW for 2 min. The root tips were crushed with 40% acetocarmine with a dissecting needle, and the coverslip was carefully placed on the slide for microscopic study. The experiment was performed in triplicate. Chromosomal abnormalities were observed under the microscope, and the mitotic index was calculated as follows.

$$\begin{array}{l} Mitotic\;index\;\left(MI\right)\;=\frac{TDC}{TC}\times 100\\ Phase\;index\;\left(PI\right)=\frac{TC}{TDC}\times 100 \end{array}$$

Where TDC—dividing cells, TC—total number of cell counted.

#### DNA Interaction Activity

Studies of DNA interaction were performed by the method described by Chandraker et al. (2019b), Different concentrations of CT-DNA (20–260 μl) in Tris-HCl buffer (pH 7.2) were treated with BP-AgNPs (10 μM) in 1 % aqueous DMSO. UV-visible spectrophotometer further examined the specific concentration of the combined solution of CT-DNA and BP-AgNPs.

#### Photocatalytic Activity

The BP-AgNPs was studied for its catalytic property under sunlight in the degradation of toxic dyes methylene blue (MB) and rhodamine-B (RB). The dye degradation experiment was performed, according to Rodríguez-Cabo et al. (2017). The dye solutions were prepared by dissolving 2.5 mg MB and RB in 250 ml DDW, separately. In both the dye solutions (50 ml), 10 mg of BP-AgNPs were added individually. A control of MB and RB were also prepared without NPs and retained under the same condition. The UV-vis spectra and absorption maxima were monitored at fixed intervals.

#### H_2_O_2_ Sensing Capability

Following the standard method of Aadil et al. (2016) with a minor alteration, the H_2_O_2_ sensing capability of BP-AgNPs has been noted. In a colloidal solution (3 mL) of BP-AgNPs, 20 mM H_2_O_2_ (1 ml) was thoroughly mixed. The initial UV-visual spectrum of BP-AgNPs solution without H_2_O_2_ has been recorded. Further, the UV-vis spectra of the reacting solution was observed at fixed intervals.

#### Antioxidant Activity

The antioxidant activity of BP-AgNPs was evaluated by the DPPH and ABTS free radical scavenging assays. By following the conventional method of Butola and Verma (2019), the DPPH and ABTS reducing potential of BP-AgNPs and the standard reference compound ascorbic acid were determined. DPPH solution (0.1 mM) in methanol was mixed with varying concentration of methanolic BP-AgNPs. After 30 min of incubation in dark, the absorbance (517 nm) was recorded. Similarly, ABTS free radical solution was prepared with potassium persulphate, and treated with different concentrations of BP-AgNPs. After 30 min of incubation, the absorbance was taken at 734 nm. The percentage scavenging activity was calculated using the following formula.

$$\begin{array}{l} \%\;Scavenging\;activity\\ =\;\frac{Absorbance\;of\;control-Absorbance\;of\;sample}{Absorbance\;of\;control}\times 100 \end{array}$$

### Statistical Analysis

Antimitotic, antioxidant and MTT assays were performed in triplicate and their data expressed in mean ± SE following OriginPro 8.5 software. For MTT assay, data were analyzed by Student's *t*-test, where *P* < 0.05 was considered statistically significant.

## Results and Discussion

### Phytochemical Analysis

**Figure 1:**
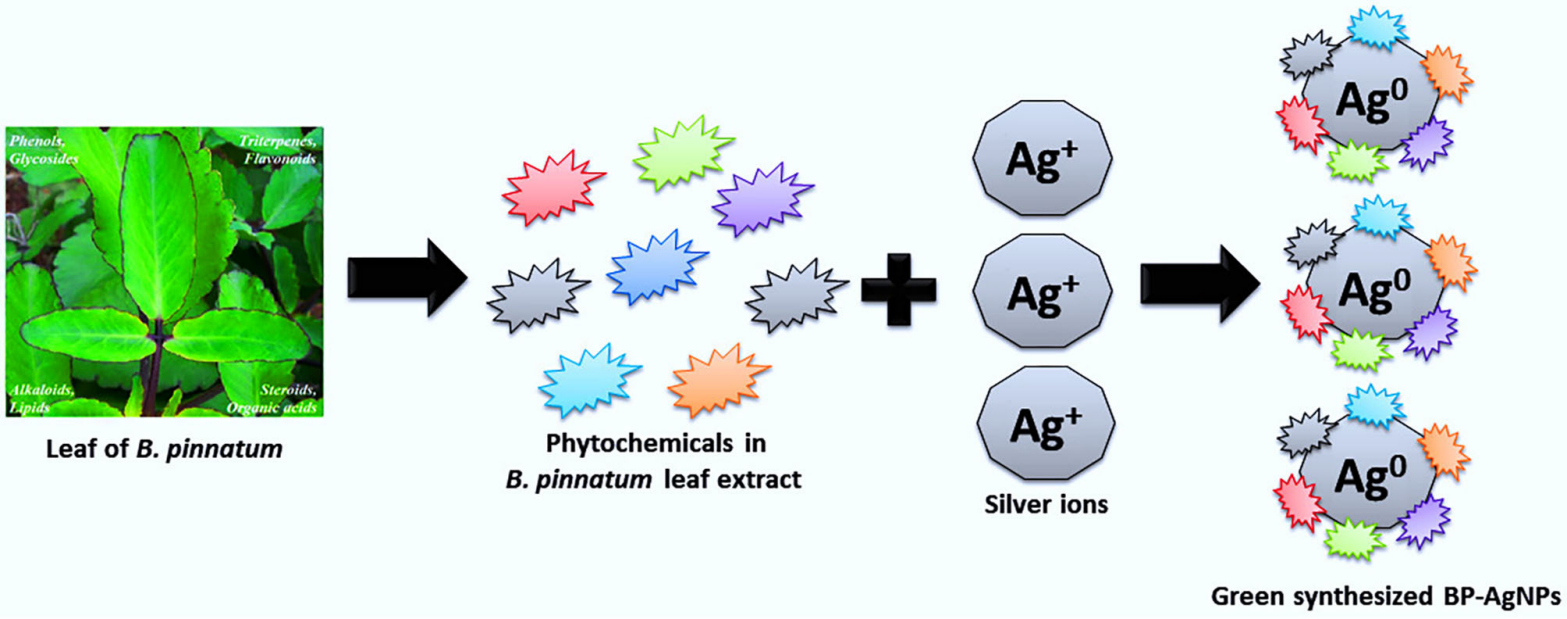
A possible mechanism for the reduction of Ag^+^ to Ag^0^ and synthesis of BP-AgNPs.

The occurrence of flavonoids, terpenoids, saponins, alkaloids, and tannins in BPLE was confirmed by various qualitative tests (Table 1). Such phytochemicals might be adsorbed on the surface of Ag+ causing their reduction to Ag^0^, and further, prevent their agglomeration. The previous studies also reported the presence of the same compounds in *B. pinnatum* (Uchegbu et al., 2017). Among several metabolites, flavonoids were explored to share a significant role in the ethnomedicinal importance of *B. pinnatum* (Chibli et al., 2014; Fernandes et al., 2019). Besides, pure flavonoids are said to be mainly compound for the transformation of metallic ions to their nanostructures with enhanced anti-microbial and anti-cancer activities (Jain and Mehata, 2017). Similarly, alkaloids, tannins, and saponins have also been reported as green reducing agents to develop novel nanomaterials with versatile applications (Ahmad, 2014; Almadiy and Nenanaah, 2018; Choi et al., 2018). The schematic of BP-AgNPs synthesis is shown in Figure 1.

**Table 1:** Phytochemical analyses of BPLE.

**S. No**.	**Phytochemicals**	**Tests performed**	**Result**
1.	Flavanoids	Ferric chloride test, Lead acetate test	+ve
2.	Alkaloids	Mayer's test, Wagner test	+ve
3.	Phytosterols	Salkowski's test, Libermann-Buchard's test	-ve
4.	Anthocyanin	NaOH test	-ve
5.	Tannins	Ferric chloride test	+ve
6.	Phlobatannins	HCL test	-ve
7.	Terpenoides	Trim-Hill reagent test	+ve
8.	Anthraquinone	Benzene test	-ve
9.	Saponins	Foam test	+ve
10.	Glycosides	Molisch test, Keller Killani test	-ve

### Green Synthesis of BP-AgNPs and Their Optimization

**Figure 2:**
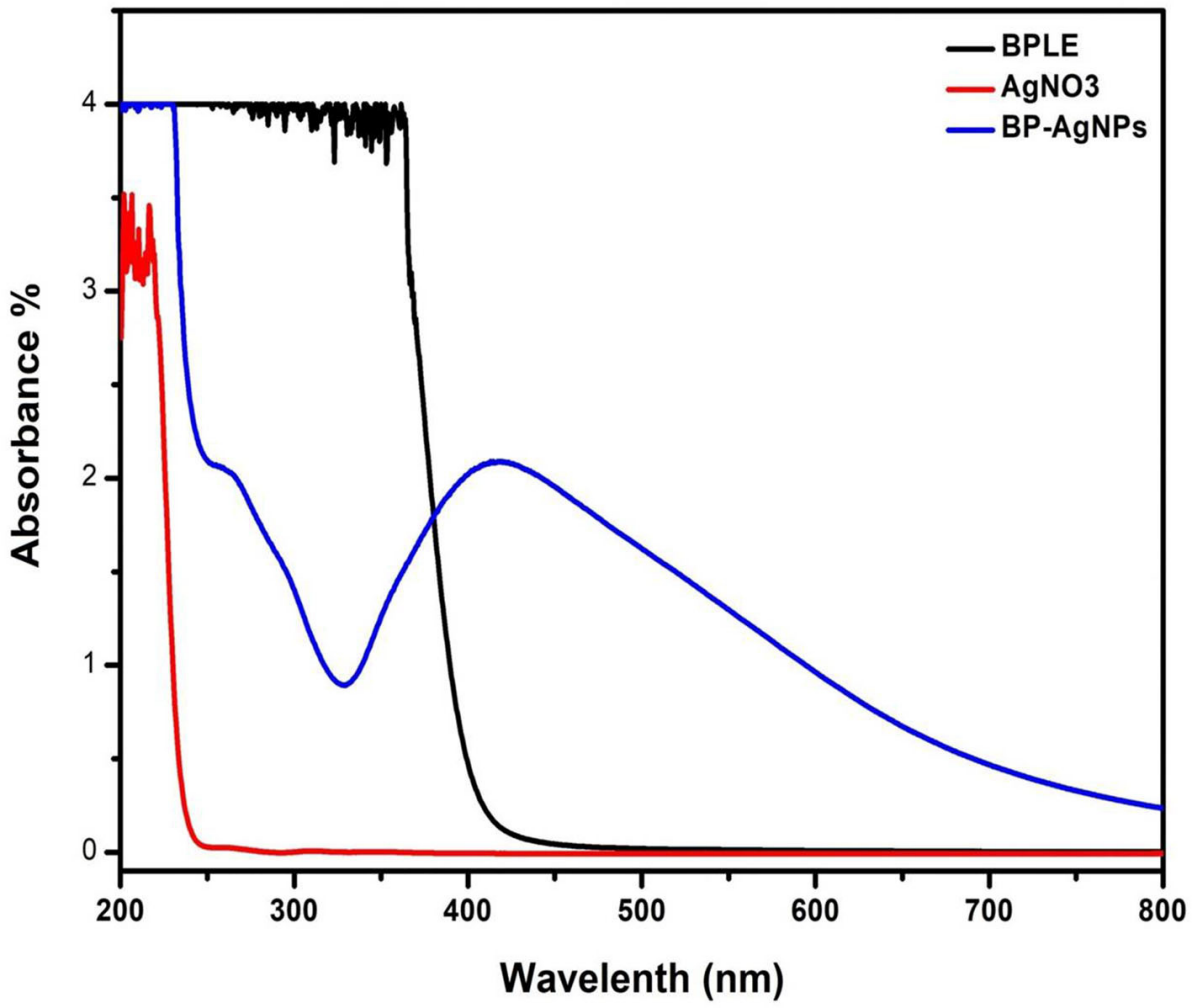
UV–Vis spectra of BPLE, AgNO_3_, and colloidal dispersion of BP-AgNPs.

UV-visible spectroscopy is a relible and basic technique for characterizing nanoparticles. Metallic NPs exhibit absorption bands in the visible region because of surface plasmon resonance (SPR) band (Lopes et al., 2018). Therefore, the synthesis of BP-AgNPs was confirmed by spectroscopic analysis through their distinctive SPR peak at 412 nm, as the band is typical of the nanoscale silver. Whereas, no absorption spectra was detected with an aqueous solution of BPLE and suspension of AgNO_3_ salt, separately (Figure 2). Green synthesis of BP-AgNP tends to be highly selective and relies on some essential factors, i.e., extract concentration, pH, temperature, and AgNO_3_ concentration. Because of their distinctive properties, these parameters regulate the size, shape, yield, stability, and agglomeration of the BP-AgNPs.

### Effect of Extract Concentration

**Figure 3:**
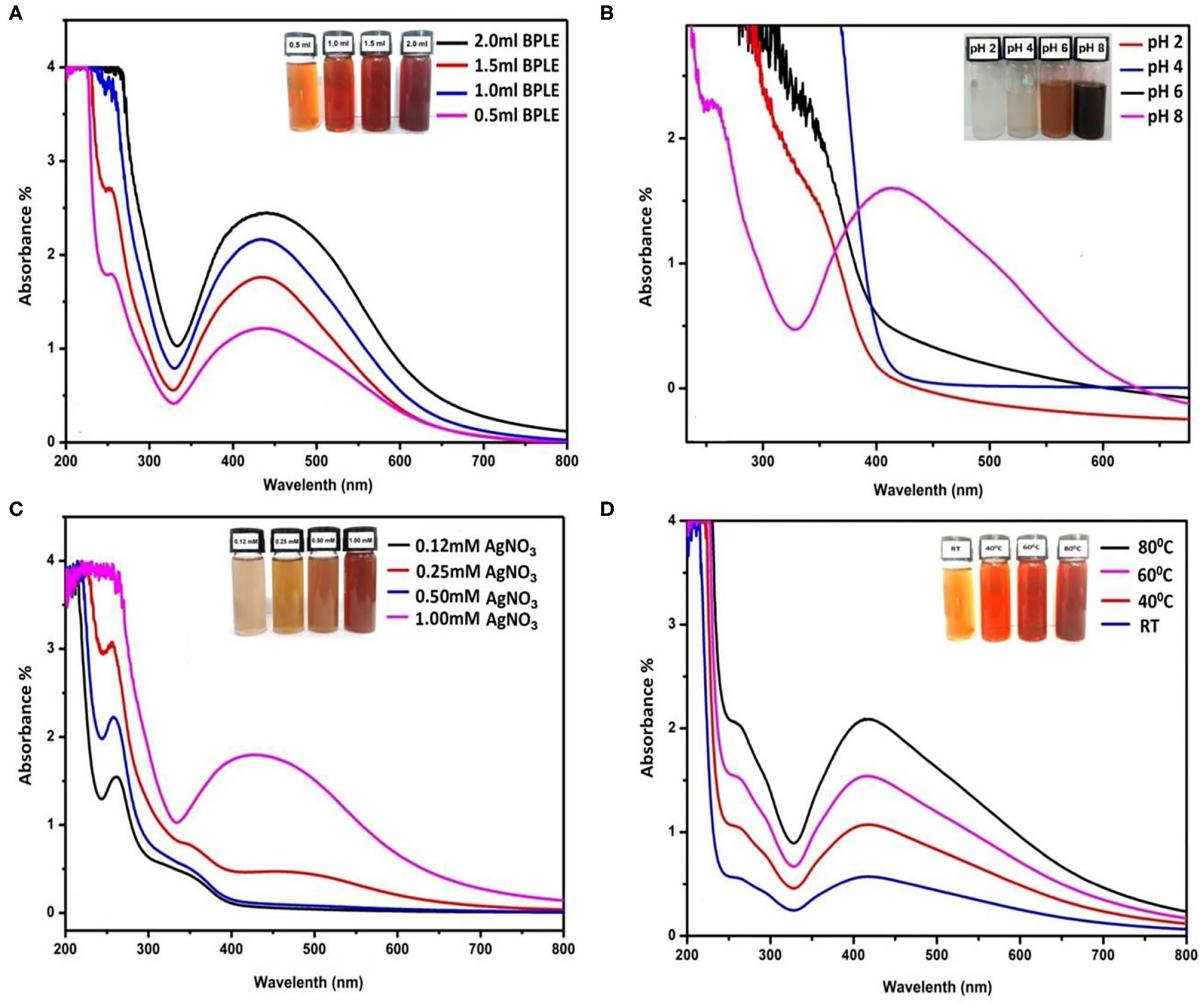
UV–Vis spectra of BP-AgNPs synthesis on variable concentrations of BPLE **(A)**; on variable pH **(B)**; on variable concentrations of AgNO_3_ solutions **(C)**, and on variable temperature **(D)**.

A concentration-dependent effect of leaf extract was found in BP-AgNPs synthesis (Figure 3A). The green synthesis of BP-AgNPs is very low at 0.5 mL concentration of BPLE. This is because of the lesser availability of phytochemicals like phenols, triterpenes, glycosides, alkaloids, flavonoids, steroids, lipids, and organic acids, which act as capping, reducing, and stabilizing agent in phytosyntesis process (Nabikhan et al., 2010). The optimal level of leaf extract was found to be 2.0 mL for green synthesis nanoparticles.

### Effect of pH

pH plays a major role in green synthesis of BP-AgNPs. It was confirmed that the size and shape of the NPs could be regulated by adjusting the pH of the solution media (Patra and Baek, 2014). In Figure 3B, the characteristic SPR peak of Ag^0^ was not observed at acidic pH. However, a slightly basic medium (pH 8) supports the formation of BP-AgNPs. The obtained result is similar to earlier observations of Gurunathan (2019) and Vanaja et al. (2013) where acidic pH either suppresses NPs formation or gives low absorbance band, whereas, negative ions (OH^−^) after dissociation of NaOH in basic medium causes much reduction of Ag^+^ into AgNPs.

### Effect of AgNO_3_ Concentration

By taking variable concentrations of AgNO_3_ in the optimization experiment, it was found that on lower concentrations (0.12–0.5 mM), the absorption peak for AgNPs was not observed (Figure 3C). This may be due to the very low availability of Ag^+^ ions in the reaction mixture. At 1.0 mM AgNO_3_, significant absorption spectra (412 nm) was found in the UV-vis spectrophotometer. Hence, 1 mM AgNO_3_ is the optimal concentration for green synthesis of AgNPs. However, in some other reports, a further increase in concentration up to 5 mM showed more absorbance (hyperchromic shift) at the same wavelength and produced larger NPs (Vanaja et al., 2013).

### Effect of Temperature

Temperature is also a relevant parameter for NPs synthesis. The physical and chemical methods require respective temperatures >350 and <350°C, whereas, green synthesis approach follows ambient to 100°C (Patra and Baek, 2014). In the present study, the synthesis of BP-AgNPs increases with increasing the reaction temperature. The UV-visible spectra (Figure 3D) showed that 60°C is the most suitable condition for BP-AgNP synthesis. At room temperatureand 40°C, synthesis of NPs is very low to moderate, whereas, at 80°C, nanoparticle synthesis is high but not favored due to aggregation. A similar result was obtained using olive leaf mediated NPs at different temperatures (Khalil et al., 2014).

The stability of BP-AgNPs was checked regularly following 8 months of green synthesis. The spectral data suggested adequate stability of NPs with a slight increase in the SPR peak, which may be due to proximity effect or agglomeration.

### FT-IR Spectral Analysis

**Figure 4:**
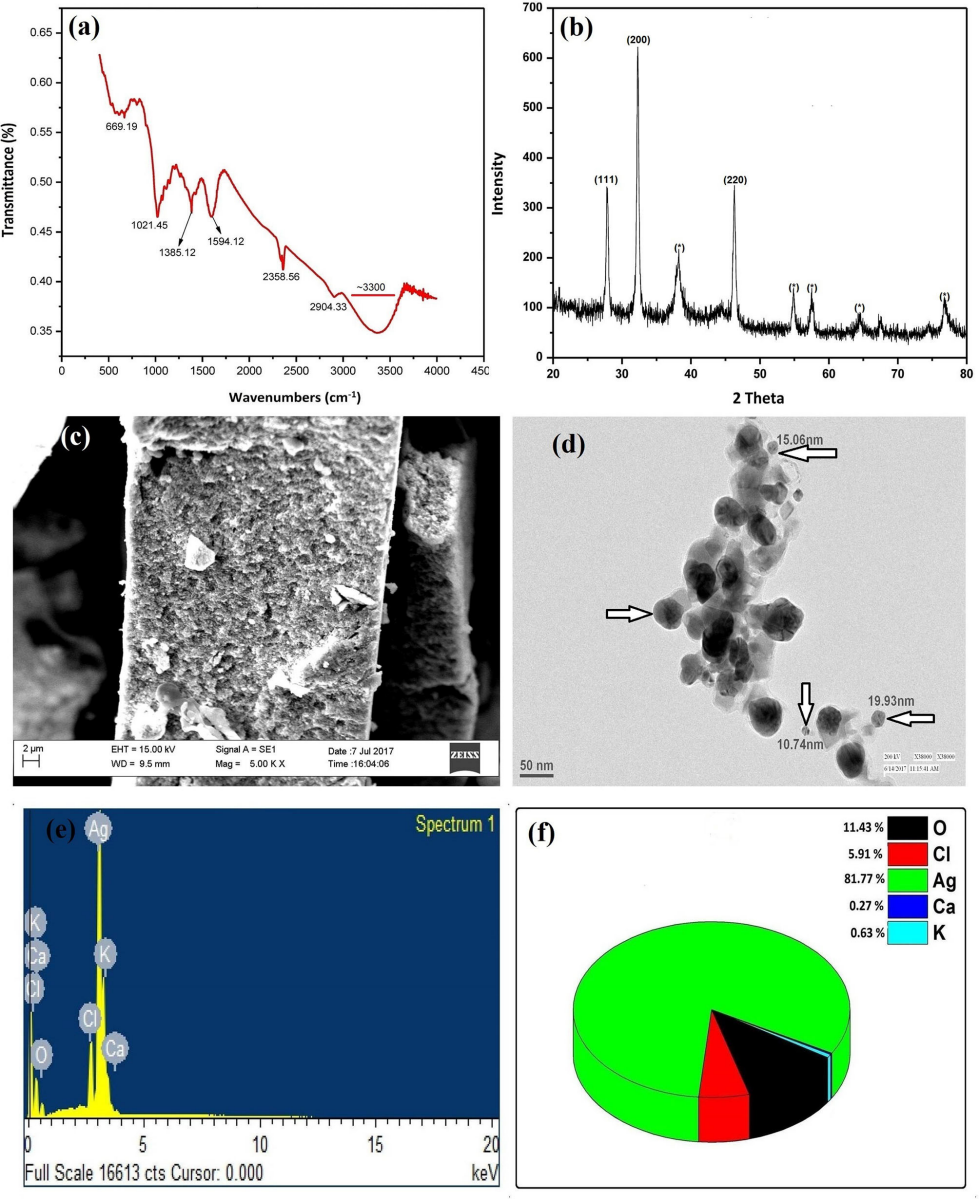
FT-IR spectra of BP-AgNPs **(a)**, XRD pattern of BP-AgNPs showing crystalline Ag facets **(b)**, SEM **(c)**, and TEM **(d)** images of BP-AgNPs, EDX spectra **(e)**, and elemental composition of BP-AgNPs **(f)**.

The FT-IR spectrum of BP-AgNPs is shown in Figure 4a, where noticed peaks denote functional groups in various chemical compounds, such as flavonoids, polysaccharides, polyphenols, and triterpenoids. The FTIR bands at 3,000–3,300, 2904.33, 2358.56, 1594.12, 1385.12, 1021.45, and 669.19 cm^−1^. The peak centered on ~3,300 cm^−1^ corresponding to O-H stretching, whereas, 2904.33 cm^−1^ suggests amide C–C stretching. The peak observed at 2358.95 cm^−1^ may be due to C–H stretching of a methylene group, and the peak at 1594.12 cm^−1^ corresponds to -C=C- and -C=N stretching. The band at 1385.12 cm^−1^ corresponds to O-H bend, and 1021.45 cm^−1^ can be attributed to the stretching vibration of the O–C bend. The final peak observed at 669.17 cm^−1^ may be due to the N–H stretching of the amide group (Nabikhan et al., 2010).

### XRD Analysis

XRD profile of phytosynthesized BP-AgNP is shown in Figure 4b, confirming the presence of Ag in the sample. In the XRD graph at 2θ = 28, 32, and 46, the Bragg reflections were observed that noticeably shows the existence of (111), (200), and (220) lattice plane, respectively. The peak pattern can be an indexed face-centered cubic (fcc) silver (JCPDS, File No. 893722) (Sudhakar et al., 2015). From the analysis of XRD Pattern it was confirmed that green synthesized particles had a nano-size with crystalline nature. Certain extra unattributed peaks near typical peaks were also recorded, showing the crystallization of the bioorganic stage on the NP's surface.

### SEM and EDX Analysis

The topology and morphology of BP-AgNPs were visualized through SEM assessment. The synthesis of consistent and comparatively orbicular BP-AgNPs is confirmed in Figure 4c. Energy-dispersive X-ray analysis (EDX) reveals the composition of elementals in BP-AgNPs. The EDX spectrum, which shows the major elementary peak at 3 keV, indicates metallic silver in Figure 4e. During the capping of AgNP by biomolecules of BPLE, other small peaks of K, Cl, Ca, and O were also created. The quantitative estimate shows that elemental Ag has a maximum weight percentage of 81.77%, while O, Cl, Ca, and K having had 11.43, 5.91, 0.27, and 0.63%, respectively Figure 4f.

### TEM Analysis

Figure 4d displays the TEM image which elucidates the formation of isotropic, nearly spherical NPs. The average particle size was measured 15 ± 5 nm, following the particle size determined from the XRD.

### *In vitro* Cytotoxic Activity

**Figure 5:**
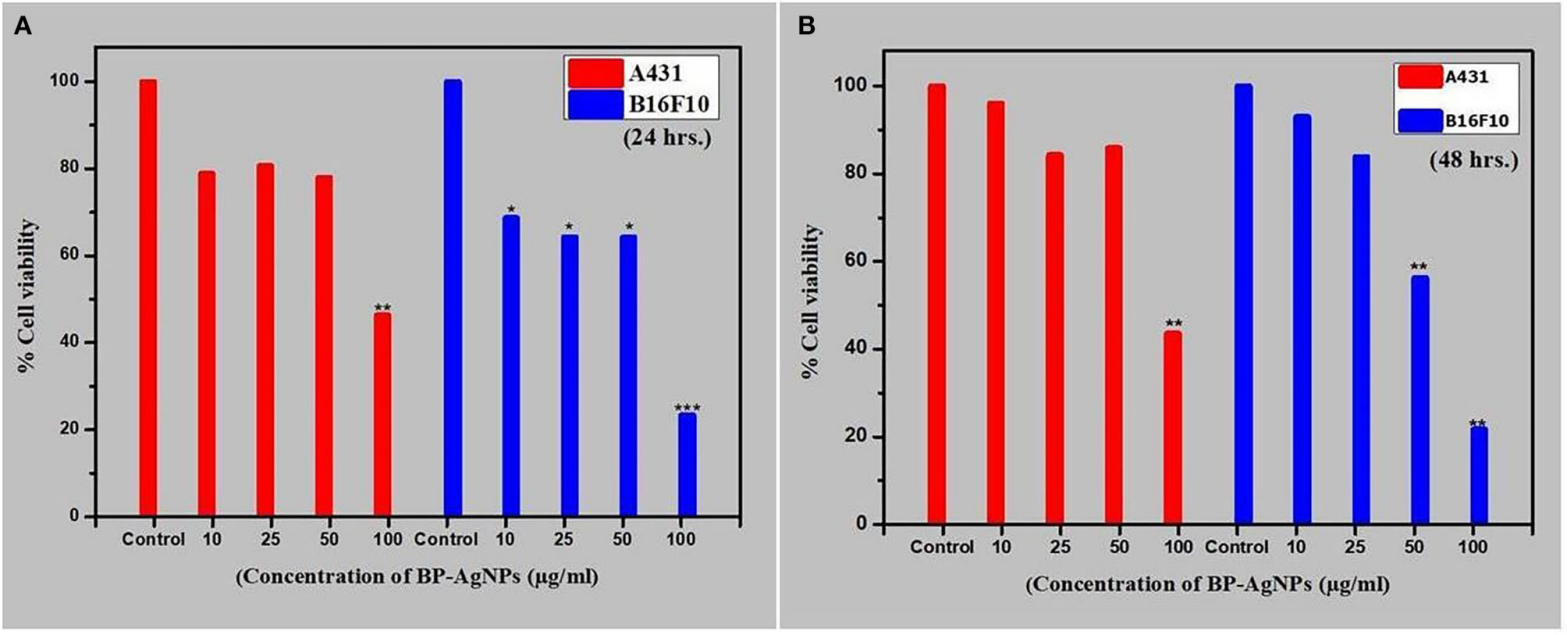
Effect of BP-AgNPson cell viability of A431 and B16F10 cells after **(A)** 24 h **(B)** 48 h of treatment. The assay was done in triplicate and the data are represented as mean ± SE; significance value *p ≤ 0.05,**p ≤ 0.01, ***p < 0.001 as calculated by Student's *t*-test.

MTT assay was performed to determine the cytotoxic property of BP-AgNPs. It is a colorimetric measurement that analyses the emergence of purple-blue formazan crystals by reduction of yellow color dye 3-(4,5-dimethylthiazol-2-yl)-2,5-diphenyl tetrazolium bromide (MTT) by the mitochondrial enzyme succinate dehydrogenase. Based on the viability of cancer cells (human squamous cell carcinoma A431 and mouse melanoma B16F10), the effect of NPs was analyzed. *In vitro* results displayed a decrease in viability of both A431 and B16F10 cells with an increase in the concentration of BP-AgNP_S_ after 24 h and 48 h of treatment. After 24 h of treatment with BP-AgNPS, there was not much decrease in cell viability at the lower concentrations of 10, 25 and 50 μg/ml, but at higher concentration of 100 μg/ml, cell viability was reduced significantly by ~50% in A431 cells (*p* < 0.01) and 80% in B16F10 cancer cells (*p* < 0.005) in comparison to untreated control cells. Similar results were found after 48 h of treatment, and there was a concentration-dependent decrease in viability of A431 and B16F10 cancer cells. Cell viability was ~46% in A431 cells (*p* < 0.01) and 23% in B16F10 cells (*p* < 0.01) compared to control. BP-AgNPs showed significant cytotoxicity toward skin cancer cells (Figures 5A,B). BP-AgNPs were found to be more effective against B16F10 cell lines with IC_50_: 59.5 μg/ml, rather than A431 cell line showing IC_50_: 96.61 μg/ml after 24 h of treatment. In earlier reports, *Butea monosperma* mediated Ag and AuNPs were found to be a biocompatible vehicle for chemotherapeutic drugs but did not exhibit cytotoxic effects against the B16F10 cell line (Patra et al., 2015). However, phytosynthesized AgNPs using *Impatiens balsamina* flowers showed less cytotoxicity and increased IC_50_ (196.5 μg/ml) than our report against B16F10 cell line (Nalavothula et al., 2015). Likewise, several research groups documented cytotoxic activity of metallic-NPs against various cancer cell lines in a dose-dependent manner, however, biocompatibility and non-toxicity against normal cells (Ribeiro et al., 2018). In separate experiments, Balasubramani et al. (2015) and Farah et al. (2016) observed a dose-dependent decrease in viability of MCF-7 breast cancer cells with 217 and 257.8 μg/ml IC_50_ when treated with *Adenium obesum* mediated AgNPs and *Antigonon letopus* mediated AuNPs, respectively. The definite mechanism of NPs' anti-cancer exercise is not yet fully presumed. However, the generation of reactive oxygen species, up-regulation of p53 protein, and expression of caspases are considered to be the major anti-cancer mechanisms (Barabadi et al., 2017). According to Rai et al. (2016) nanoparticle generated ROS promotes caspase-3 activation that is responsible for cell apoptosis by arresting the G2/M phase of the cancerous cell cycle. Additionally, increased oxidative stress causes oxidation of antioxidant glutathione to glutathione disulfide. Hence, ROS damages not prevented in the cells (Ovais et al., 2017). Besides, AgNPs have shown to downregulate the action of DNA-dependent protein kinase, a damage repair enzyme. Jeyaraj et al. (2013) reported that AgNPs mediated Bcl-2 and Bax gene regulation, which further activates the cascade and controls the caspases 3, 8, and 9 are responsible for the apoptosis of HeLa cell line.

### Impact on Mitosis

**Figure 6:**
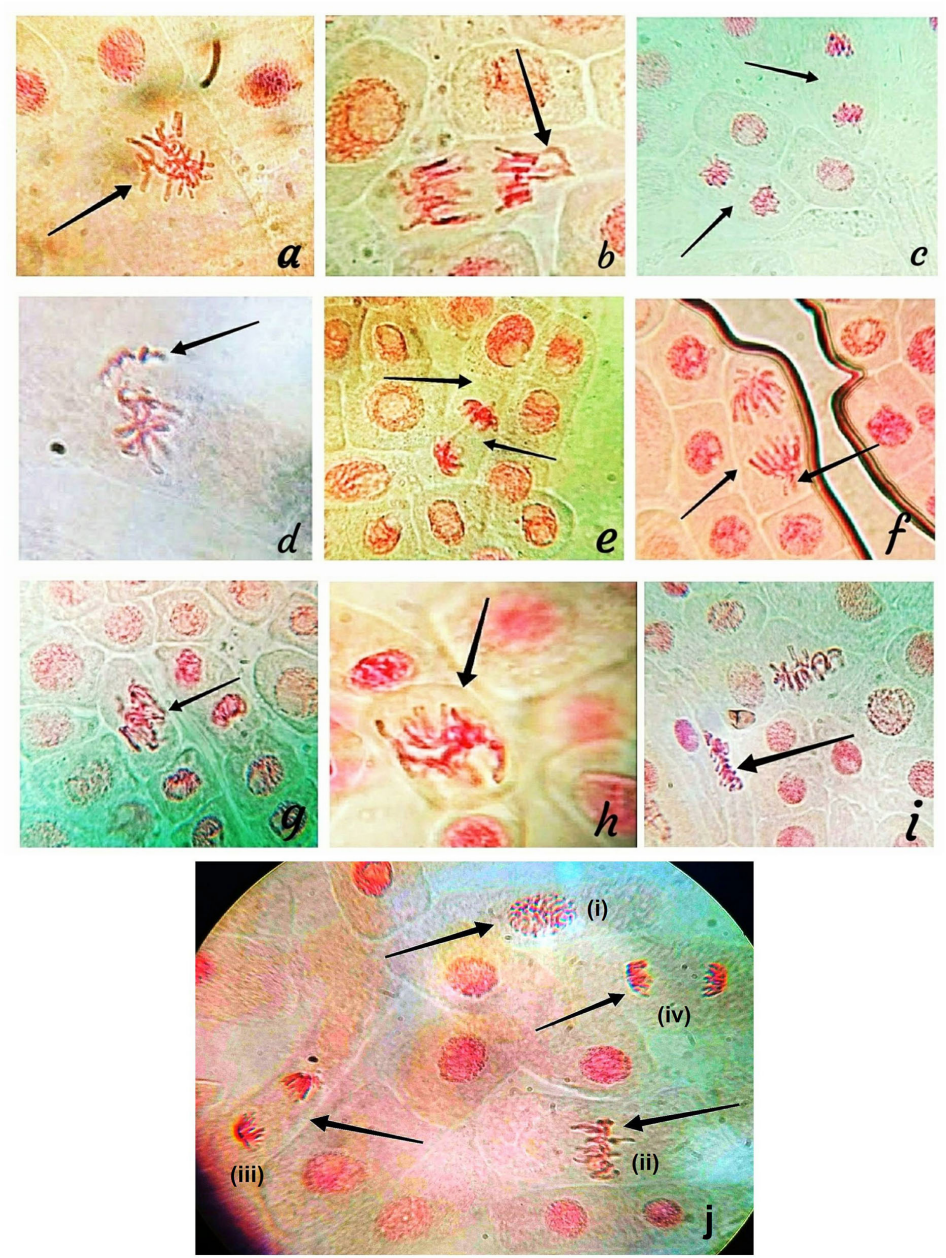
Chromosomal abnormalities: **(a)** C-metaphase, **(b)** Anaphase with lagging chromosome, **(c)** Stickiness telophase, **(d)** Vagrants chromosomes, **(e)** Anaphase with chromosome bridge, **(f)** Anaphase with lagging chromosome, **(g–i)** Sticky metaphase, **(j)** Normal mitosis: (i) Prophase, (ii) Metaphase, (iii) Anaphase and (iv) Telophase.

Allium test is suggested by UNEP, IPCS (International Programme on Chemical Safety), IPPB (International Programme on Plant Bioassay), and WHO as a typical test in cytogenetic monitoring of environmental hazards (Maity et al., 2020). The experiment is also suggested to evaluate the genotoxic effects of novel nanomaterials and considered as a substitute to animal tests (Klančnik et al., 2011). Although severalphysico-chemical mutagens, metal-complexes, and Cu, CdS-NPs have been reported to induce chromosomal (Kumbhakar et al., 2016; Sharma et al., 2018), the cytotoxic effects of Ag-NPs are poorly reported. Root meristem of *A. cepa* was used to detect the cytotoxic effect of BP-AgNP_S_ at three different concentrations (10, 20, and 30 μg/mL). The present investigation showed that BP-AgNPs decrease the mitotic index (MI) (Table 2) and induce significant alterations in the treated roots relative to the control samples (DDW and AgNO3 treated roots). BP-AgNPs might have been prompted cytotoxicity in treated roots of meristematic cells, causing DNA damage or cell death (Lateef et al., 2016). The values of MI for BP-AgNPs were found 45.0 ± 0.28, 41.66 ± 0.44, and 39.96 ± 0.08 after 12 h which significantly decreased to 40.63 ± 0.27, 38.70 ± 0.60, and 34.53 ± 0.26 after 24 h at 10, 20, and 30 μg/ml, respectively. However, in the control set and AgNO_3_ solution (30 μg/ml), the MI was found to be 55.6 ± 0.4 and 52.76 ± 1.38, respectively. The experiment demonstrated that the cell division was inversely proportional to the BP-AgNP concentrations. The findings were statistically significant (*p* < 0.05) in all the treated concentrations as compared to the control. Chromosomal abnormalities were severely observed in treated meristematic cells with C-metaphase, anaphase with lagging chromosome, stickiness telophase, vagrants chromosomes, anaphase with chromosome bridge, anaphase with lagging chromosome, sticky metaphase, etc. (Figure 6). Our observations are supported by earlier findings where NPs decrease MI with the increase in mitotic anomalies in *Allium cepa* (Nagaonkar et al., 2015; Kumbhakar et al., 2016). Few reports advocate that AgNPs might interfere with the normal cycle of mitotic cells causing decreased gene expression encoding cyclin-dependent kinase 2, slower advancement of cells to S—phase, and blockage of G2—phase, leading to cell death. AgNPs are found to alter cytoplasm viscosity which leads to atypical behavior of the spindle causing chromosomal abnormalities and formation of micronuclei (Daphedar and Taranath, 2018; Fouad and Hafez, 2018). NPs are also responsible for the breakage and reunion of chromosomal material resulting in structural and numerical changes in chromosomes (Chromosomal abbreviations).

**Table 2:** Mitotic index (MI) and phase index (PI) of *A. cepa* root meristematic cells treated with different BP-AgNP concentrations.

**Treatment**	**Concentrations (μg/ml)**	**Total no. of dividing cells**	**P**	**M**	**A**	**T**	**(MI) Mean ± SE**	**PI %**
Control (DDW) for 24 h	0.0	556	380.66	84.66	64	26.66	55.6 ± 0.41	179.8
AgNO_3_ for 24 h	30.0	527.66	372.33	69.33	60	26	52.76 ± 1.38	189.75
BP-AgNPs for 12 h	10.0	450	308.33	68	59.66	14	45.0 ± 0.28	222.22
	20.0	416.66	309	50.66	40	17	41.66 ± 0.44	240.38
	30.0	399.66	305	47	34	16.33	39.96 ± 0.08	250.62
BP-AgNPs for 24 h	10.0	406.33	308.66	45.33	36.33	16	40.63 ± 0.27	246.30
	20.0	387	297.33	44.33	34.33	11	38.70 ± 0.60	258.39
	30.0	345.33	281.33	32.33	22.66	10.66	34.53 ± 0.26	289.85

*Where P, M, A, and T are prophase, metaphase, anaphase, and telophase, respectively*.

### DNA Interaction Study

**Figure 7:**
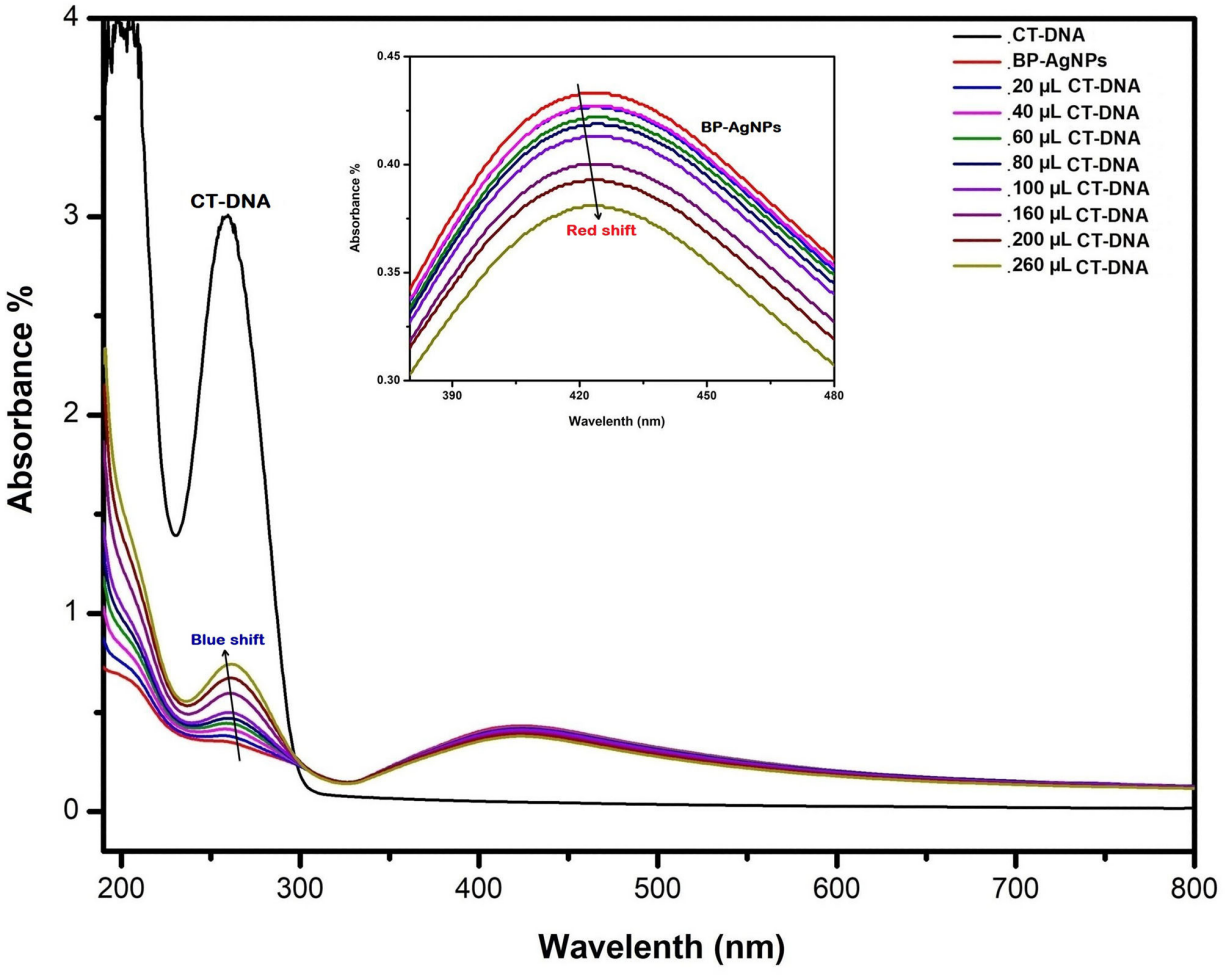
UV-Vis absorption spectra of BP-AgNPs after treatment with various concentrations of CT-DNA (20–260 mL).

The UV-vis spectroscopy is one of the most significant techniques for determining the efficiency of the compounds to interact with DNA. Before the addition of BP-AgNP, the purity and sustainability of CT-DNA were tested at room temperature showing a high absorption peak at 264 nm. The concentration of BP-AgNPs was made constant and CT-DNA was added gradually for the study. Figure 7 displayed a decrease in the absorption of NPs (i.e., hypochromic) with a minor bathochromic or red-shift in spectra (420–424 nm) preferably indicating intercalation of BP-AgNPs with the corresponding DNA (Komal and Kaushik, 2019). The absorption spectra of CT-DNA demonstrated a minor blue shift or hypsochromic effect (264–260 nm) with the isosbestic point showing a strong interaction of NPs with CT-DNA. There are a few reports which have mentioned such kind of interaction between nanostructures and DNA as providing such an interactive feature. Three possible modes for these interactions are electrostatic linking, groove linking, and intercalation (Ribeiro et al., 2018).

### Photocatalytic Activity

**Figure 8:**
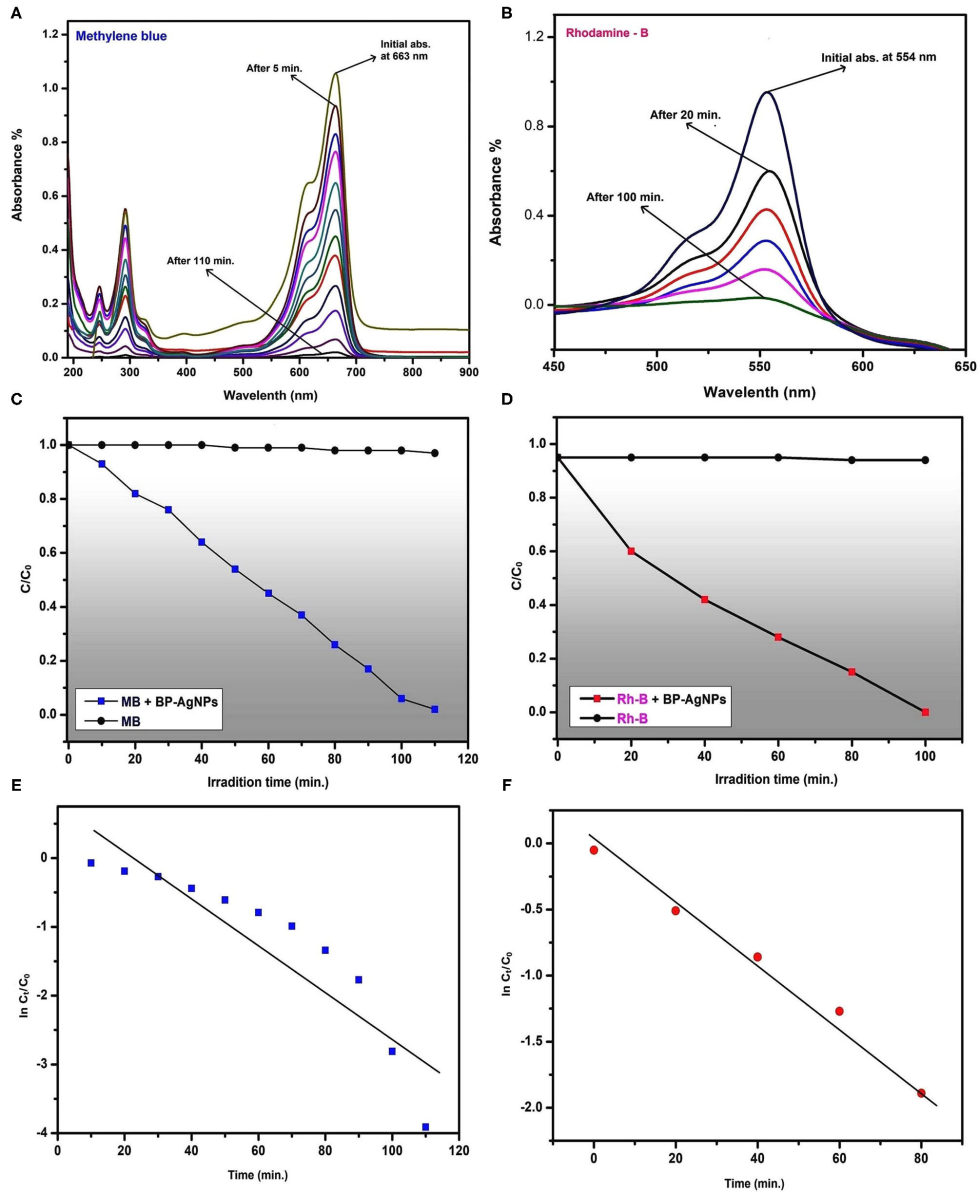
UV-Visible spectra recorded at regular intervals displaying gradual reduction of MB **(A)** and Rh-B **(B)**, The changes in concentrations of MB **(C)** and Rh-B **(D)** in presence of BP-AgNPs and sunlight, Plot of ln(C_t_/C_0_) vs. time (min) for photocatalytic degradation of MB **(E)** and Rh-B **(F)**.

Two different types of dyes (thiazine: MB and azo: Rh-B) were selected as model pollutants to study the dye degradation capabilities of BP-AgNPs under solar irradiation. Both the dyes are positively charged (basic). The distinctive absorption maxima for MB and Rh-B were peaked at 663 and 554 nm, respectively (Figures 8A,B). MB and Rh-B are gradually degraded which is expressed by a significant decrease in peak strength with time increase. Until the end of the exposure period, there was no color change in the control set of both the dyes in the absence of BP-AgNPs (Figures 8C,D). The efficacy of BP-AgNPs in dye degradation was determined as follows (Alshehri et al., 2017).

$$\begin{array}{l} Dye\;degradation\;\left(\%\right)=\left[1-\;\frac{C_{t}}{C_{0}}\right]\times 100 \end{array}$$

Where C_0_ is the initial concentration of the dye solution at t = *0* and Ct is the concentration of the dye solution after a specific exposure to sunlight. In the present work, it is represented in plot that MB and Rh-B were degraded 100% within 110 and 100 min, respectively. However, in similar reports, MB was found to be degraded significantly, after 6 and 72 h from *Cordia dichotoma* and *Morinda tinctoria* mediated NPs, respectively (Vanaja et al., 2014; Kumari et al., 2016). Green synthesized NPs from *Zanthoxylum armatum* were also reported for noteworthy degradation of toxic dyes Safranine-O, Methyl-red, Methyl-orange, and MB after 24 h (Jyoti and Singh, 2016). Thus, the biofabricated BP-AgNPs can serve as a stable and effective green catalyst for the nano-degradation of MB and Rh-B under visible light.

The kinetic study is among the most relevant approaches from which reaction mechanisms are described. In Figures 8E,F, a linear plot (with slope: −0.022 for MB and −0.033 for Rh-B) of ln(C_t_/C_0_) vs. time for photocatalytic degradation of MB and Rh-B by BP-AgNPs, shows pseudo-first -order kinetics. The rate constant value of MB degradation is calculated 4.9 × 10^−4^ sec^−1^ and for Rh-B 4.5 × 10^−4^ sec^−1^. The regression coefficient (for MB R2 = 0.98 and Rh-B R2 = 0.80) revealed that the degradation rate of MB and Rh-B were following the Langmuir-Hinshelwood kinetic model.

### H_2_O_2_ Sensing Capability

**Figure 9:**
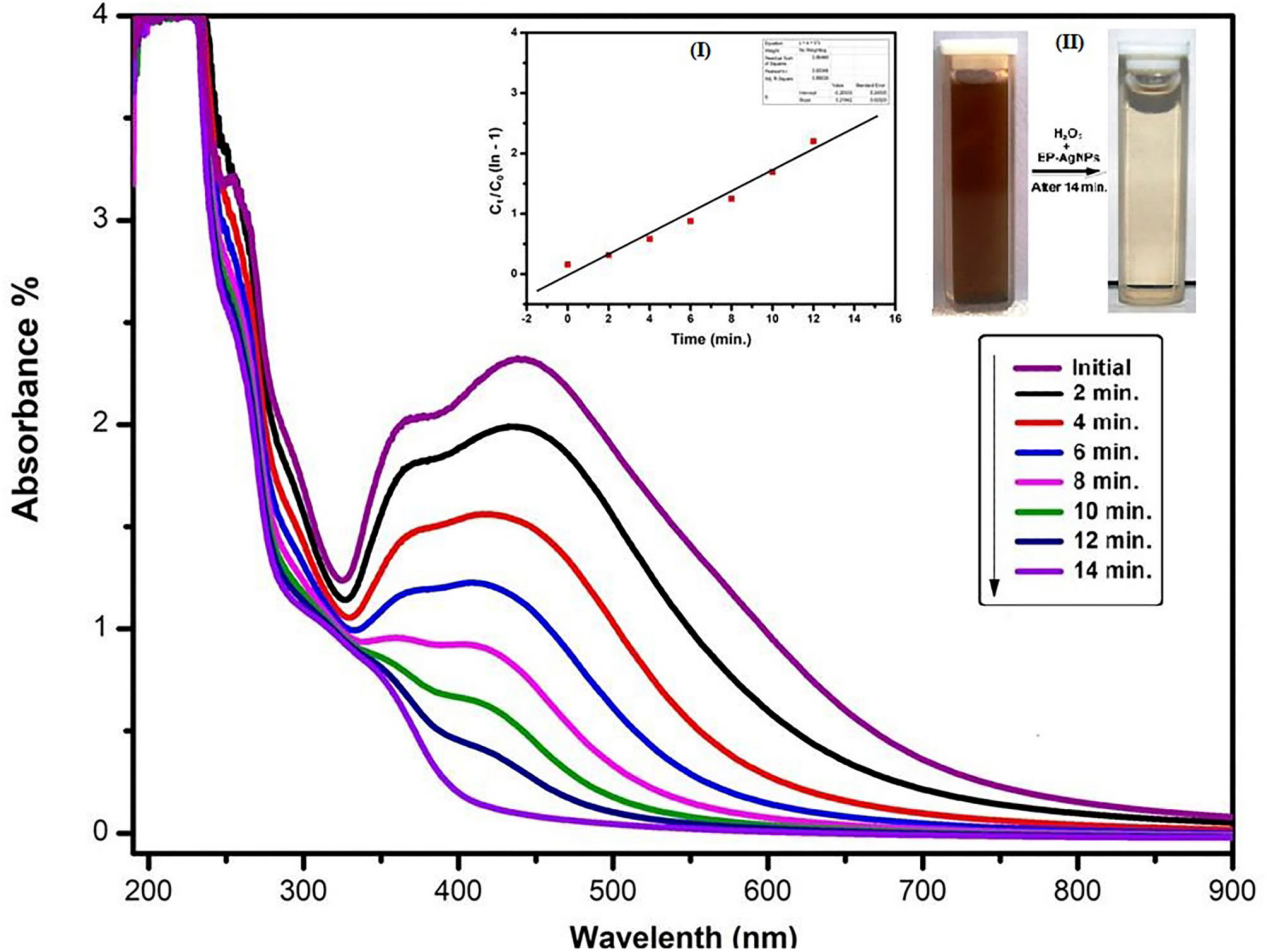
UV-Vis absorption spectra of BP-AgNPs observed at regular interval after the addition of H_2_O_2_, [Inset: (i) Reacting solution after 14 min and (ii) plot of C_t_/C_0_ (1- ln) versus time (min) for H_2_O_2_ sensing by BP-AgNPs].

BP-AgNPs was evaluated with potential H_2_O_2_ sensing capacity in the present report. The comparative spectra at regular interval is shown in Figure 9. The brown color (vial a) constantly diminished and finally appeared colorless (vial b) after 14 min (Figure 9ii). In the control set without H_2_O_2_, no changes in the color and intensity of the SPR absorbance were observed. Mohan et al. (2014) proposed that the inclusion of AgNPs to H_2_O_2_ causes the creation of reactive oxygen species, that trigger the degeneration AgNPs. Figure 9(i) indicates a linear plot (slope: 0.021) of C_t_/C_0_ (1-ln) vs. time for BP-AgNPs mediated H_2_O_2_ sensing, exhibiting pseudo-first-order kinetics. The sensing rate constant value for H_2_O_2_ is estimated at 3.8 × 10–3 s^−1^. The regression coefficient of H_2_O_2_, R^2^ = 0.88 indicated that the sensing capacity of BP-AgNPs has followed the kinetic model of Langmuir—Hinshelwood.

These results indicate that BP-AgNPs can be used successfully to identify H_2_O_2_ concentration in several unknown samples or environmental effluents. Similar NPs-based H_2_O_2_ sensors were also reported using *Bacillus subtilis* and *Calliandra haematocephala* (Mohan et al., 2014; Raja et al., 2017).

The following mechanism shows the probable H_2_O_2_ decomposition reaction process by BP- AgNPs (Ghosh et al., 2019).

$$\begin{array}{l} {\text{H}}_{2}{\text{O}}_{2}⇌2{\text{H}}^{+}+{\text{H}}_{2}\text{O}\\ \text{BP}-{\text{Ag}}^{0}\text{NPs}⇌\left[\text{BP}-{\text{Ag}}^{1}\text{NPs}\right]+{\text{H}}^{+}\\ \left[\text{BP}-{\text{Ag}}^{1}\text{NPs}\right]+{\text{H}}^{+}+{\text{HO}}_{2}^{-}⇌{\left[\text{BP}-{\text{Ag}}^{1}({\text{HO}}_{2})\text{NPs}\right]}^{-}\\ {\left[\text{BP}-{\text{Ag}}^{1}({\text{HO}}_{\text{2}})\text{NPs}\right]}^{-}⇌{\left[\text{BP}-{\text{Ag}}^{0}(\text{HO}{*}_{2})\text{NPs}\right]}^{-}\\ \text{Intermediated }(\text{active species})\\ {\left[\text{BP}-{\text{Ag}}^{0}(\text{HO}{*}_{2})\text{NPs}\right]}^{-}⇌{\left[\text{BP}-{\text{Ag}}^{0}{\text{O}}_{2}\text{NPs}\right]}^{-}\\ \text{Rate}=\frac{{\text{K}}_{1}\sqrt{{\text{K}}_{1}}{\text{K}}_{2}{\text{K}}_{3}\text{BP}-{\text{Ag}}^{0}(\text{HO }*\;2)\text{NPs})}{\left[{\text{H}}^{+}\right]} \end{array}$$

### Antioxidant Activity

**Figure 10:**
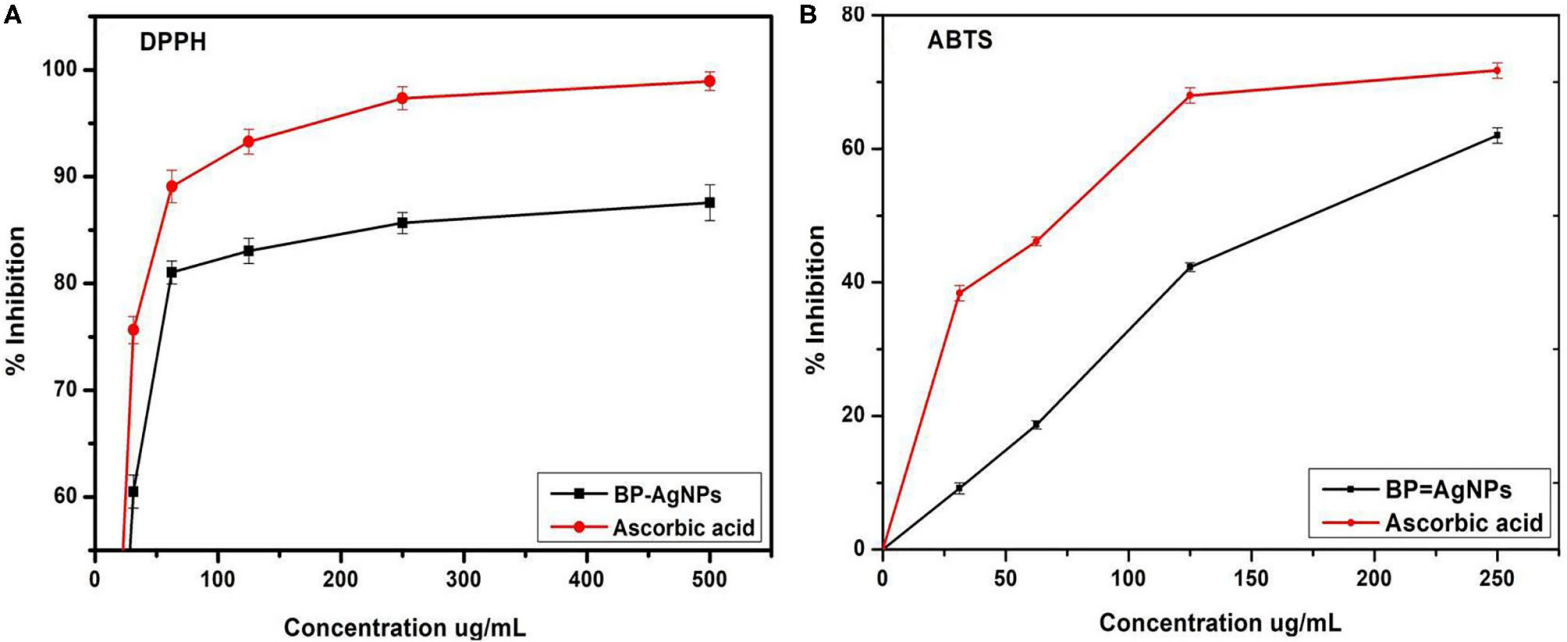
DPPH **(A)** and ABTS **(B)** free radical scavenging activity of BP-AgNPs.

DPPH and ABTS assays are relatively quick and sensitive techniques used to analyze the antioxidant activity of different compounds. DPPH is a strong free radical that transforms into a diamagnetic compound (bright yellow) when counter with electron or hydrogen molecules. The magnitude of the color transition of DPPH from violet-blue to bright-yellow depends on the nature and amount of the substance. Figures 10A,B are showing dose-dependent free radical scavenging activity of BP-AgNPs. The concentration of BP-AgNPs ranging from 31.25 to 500 μg/mL showed 60.50–87.57% DPPH scavenging activity with 89.05 μg/mL IC_50_. However, for the ascorbic acid 75.64–98.94% scavenging activity reported with 41.5 μg/mL IC_50_ at the same concentrations (Figure 10A). Similarly, ABTS free radical scavenging was observed from 9.16 to 79.32% at 31.25–500 μg/mL BP-AgNPs with an IC_50_ value of 259.14 μg/mL, whereas, ascorbic acid displayed 38.39–88.09% inhibition with 173.12 μg/mL IC_50_ (Figure 10B). Similar observations were found with leaf extracts of *Prunus japonica* and *Desmostachya bipinnata* showing antioxidant activity in terms of free radical inhibition (Saravanakumar et al., 2017; Guntur et al., 2018). The results firmly advocate the utilization of BP-AgNPs as natural antioxidants to preserve health against oxidative stresses affiliated with degenerative diseases.

## Conclusions

Biofabrication of silver nanoparticles was successfully achieved using BPLE in the present report. A facile, rapid, and ecofriendly green synthesis approach was adopted to get stable and non-aggregated BPAgNPs taking benefit of natural capping, reducing, and stabilizing agents in the form of flavonoids, alkaloids, terpenoids, saponins and tannins present in the plant extract. FTIR spectra revealed the biomolecules adsorbed on the surface of NPs which are responsible for reducing Ag^+^ to Ag^0^ in the form of NPs. UV-visual spectroscopy, XRD, SEM, EDX, and TEM revealed the salient SPR peak at 412 nm, face-centered cubic structure, dispersion, elemental composition, and size of BP-NPs, respectively. The outstanding photocatalytic degradation of hazardous dyes (MB, RB), and H_2_O_2_ sensing abilities of BP-AgNPs make them suitable agents for nanoremediation of industrial effluents and reactive oxygen species. The biofabricated BP-AgNPs have shown *in-vitro* antioxidant activity, *in-vitro* interaction with CT-DNA, induced the chromosomal aberration in the mitotic cells of *Allium cepa*, and cytotoxic activity against A431 (squamous cell carcinoma) and B16F10 (melanoma) cell line. In our knowledge, this is the first report on various environmental and biological applications of *B. pinnatum* mediated NPs. Our results suggest potential uses of BP-AgNPs in cell biology. Similarly, The results described here provide the framework for the future implementation of AgNPs in the treatment of skin cancer. The corresponding results of the synthesized BP-AgNPs are related to the decreased expense of a process and the fact that it is a “friendly” biological synthesis method. However, more research is needed to modify the size and shape of metallic NPs.

## Data Availability Statement

The original contributions presented in the study are included in the article/supplementary materials, further inquiries can be directed to the corresponding author/s.

## Author Contributions

The BP-AgNPs was synthesized by SC. The applications of the AgNPs were investigated by SC and ML. The cytotoxicity experiment was performed and reviewed by PD and RPS. The manuscript was written and reviewed by SC and RS. All the experiments were supervised under RS. All authors contributed to the article and approved the submitted version.

## Conflict of Interest

The authors declare that the research was conducted in the absence of any commercial or financial relationships that could be construed as a potential conflict of interest.
